# Experimental Investigation on the Impact Resistance of Carbon Fibers Reinforced Coral Concrete

**DOI:** 10.3390/ma12234000

**Published:** 2019-12-02

**Authors:** Bing Liu, Jingkai Zhou, Xiaoyan Wen, Jianhua Guo, Xuanyu Zhang, Zhiheng Deng, Huailiang Wang

**Affiliations:** 1 College of Civil Engineering and Architecture, Guangxi University, Nanning 530004, China; gxulb@alu.gxu.edu.cn (B.L.);; 2 Key Laboratory of Disaster Prevention and Structural Safety of Ministry of Education, Nanning 530004, China

**Keywords:** coral concrete, carbon fibers, impact resistance, drop-weight impact test, Weibull distribution

## Abstract

In this study, the impact resistance of coral concrete with different carbon fiber (CF) dosages subjected to drop-weight impact test was investigated. For this purpose, three concrete strength grades (C20, C30, C40) and six CF dosages (0.0%, 0.3%, 0.6%, 1.0%, 1.5%, and 2.0% by weight of the binder) were considered, and a total of 18 groups of carbon fibers reinforced coral concrete (CFRCC) were cast. For each group, eight specimens were tested following the drop-weight impact test suggested by CECS 13. Then, the two-parameter Weibull distribution theory was adopted to statistically analyze the variations in experimental results. The results indicated that the addition of CFs could transform the failure pattern from obvious brittleness to relatively good ductility and improve the impact resistance of coral concrete. Moreover, the impact resistance of CFRCC increases with the CF dosage increasing. The statistical analysis showed that the probability distribution of the blow numbers at the initial crack and final failure of CFRCC approximately follows the two-parameter Weibull distribution.

## 1. Introduction

The ocean is an essential space for the sustainable development of whole humans due to its abundant resources [1]. Recently, with the rapid development of society, the development and utilization of marine resources and the development of marine industry have received extensive attention [2,3]. Therefore, there are more and more island construction projects, which have led to a significant increase in demand for marine concrete [4,5]. In addition, the utilization of locally available resources on islands as materials to mix concrete has essential practical significance because it can solve the shortage of construction materials problem, shorten the construction period, and reduce costs for distant island reef construction projects [1,6,7].

On the tropic islands, there are abundant coral reef resources [8]. Thus, the coral reef is a desired material for mixing marine concrete. Extensive researches have shown that it is feasible to use coral as the raw material of marine concrete [4,6,9,10,11]. Researchers call this concrete, which uses coral as aggregates, coral concrete [4,6,12]. Many researchers have conducted research on the various properties of coral concrete, such as the compressive strength [13,14,15], the tensile strength [3,5,16], the elastic modulus [17,18,19], the durability [12,18,20], etc. However, the impact performance of coral concrete under impact loading has been rarely studied [21].

The impact resistance is recognized today as one of the significant properties of concrete used for civil engineering [22]. Many concrete elements may be subjected to low-velocity impact loads such as road pavements, breakwater, and precast concrete piles [21,23]. Therefore, it is especially important to understand and improve the impact resistance performance of coral concrete. Some studies indicated that the addition of fibers (such as steel fibers, polypropylene fibers, carbon fibers) could improve the impact resistance of concrete [24,25,26,27]. For example, Mastali et al. [27] found that, when incorporating carbon fibers (CFs) with a length of 30 mm and a volume fraction of 2.0%, the impact resistance of concrete at initial crack and ultimate crack can be increased to 3 and 5 times that of the reference specimen, respectively. Recently, the application of CFs in concrete is more and more extensive due to its high corrosion resistance, low density, high tensile strength, and high elastic modulus [28,29,30,31]. Thus, in the present study, CFs are chosen as the enhancement material to enhance the impact resistance of coral concrete.

Several test methods, including explosive test, projectile test, Charpy pendulum test, split Hopkinson pressure bar (SHPB) test, and drop-weight impact test, have been suggested to study the impact resistance performance of fibers reinforced concrete [24,32,33,34,35]. Among them, the explosive test and projectile test are usually used for high-velocity impact test; the Charpy pendulum test, split Hopkinson pressure bar (SHPB) test, and drop-weight impact test can be used for low-velocity impact test, but the test devices for Charpy pendulum test and SHPB test are expensive, and the test steps are also complicated; the device for drop-weight impact test is not expensive and the test steps are also simple. Thus, the drop-weight impact test method has been widely adopted to low-velocity impact experiments by many researchers [24,26,36]. Thus, the drop-weight impact test method is also selected as the test method for studying the impact resistance performance of carbon fibers reinforced coral concrete (CFRCC) in this study.

The purpose of the present study is to investigate the impact resistance performance of CFRCC under impact loading. For this purpose, a total of eighteen CFRCC mixtures with three strength grades (C20, C30, C40) and six CF dosages (0.0%, 0.3%, 0.6%, 1.0%, 1.5%, 2.0% by weight of the binder) were designed. Through the drop-weight impact test, the failure patterns, the blow numbers and impact energy at the initial crack and final failure of CFRCC were obtained. Based on the experimental results, the effect of CFs and concrete strength grade on the impact resistance of CFRCC was analyzed. Moreover, a statistical analysis was conducted to analyze the experimental results by the two-parameter Weibull distribution theory. The results of this study help extend the use of CFRCC and further understanding of the nature of the impact behavior of coral concrete.

## 2. Materials and Methods

### 2.1. Raw Materials

**Figure 1:**
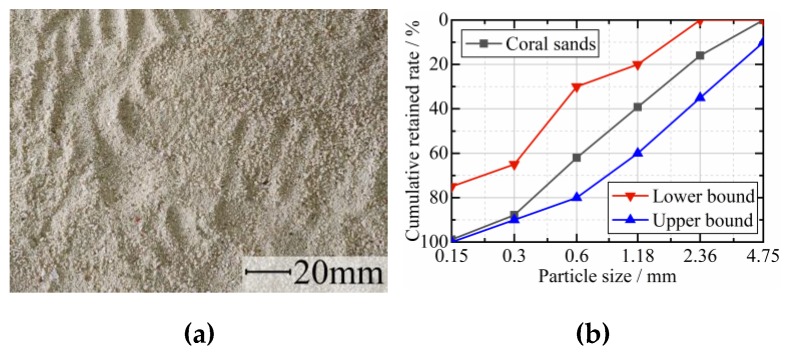
Coral fine aggregates: (**a**) appearance; (**b**) grading curve.

**Figure 2:**
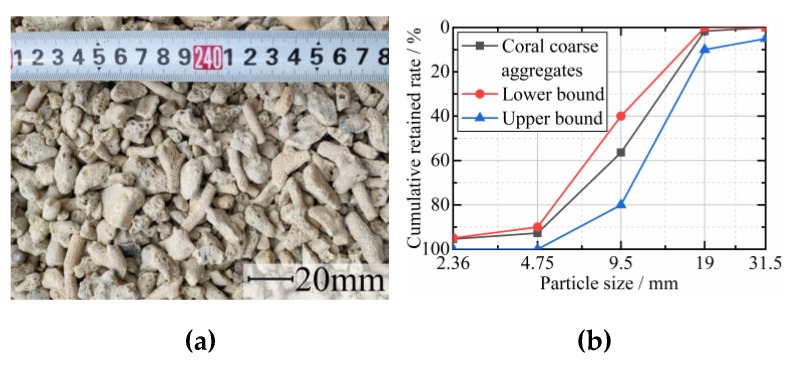
Coral coarse aggregates: (**a**) appearance; (**b**) grading curve.

**Figure 3:**
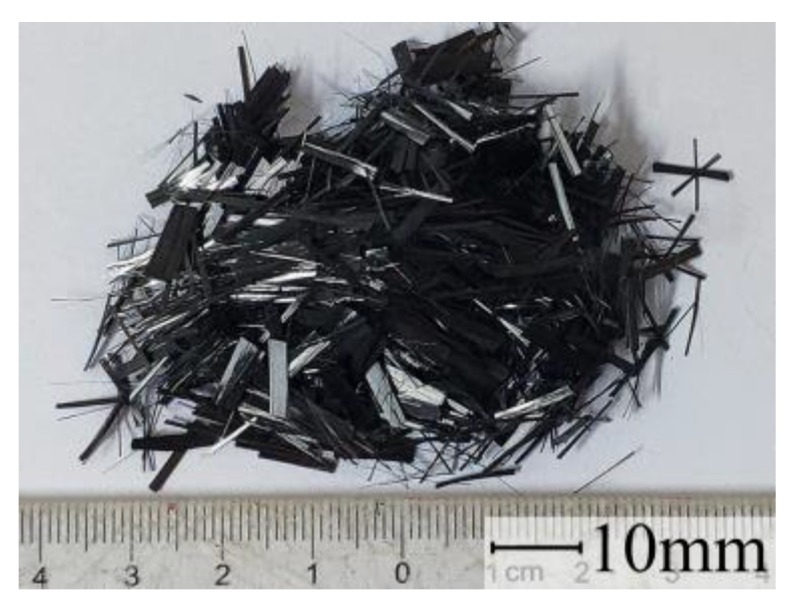
Chopped carbon fibers.

The binder was GB175 [37] Ordinary Portland P.O. 42.5 cement. Coral sand with a fineness modulus of 3.0 was used as the fine aggregates (Figure 1), while crushed coral stones (Figure 2) were used as coarse aggregates. Table 1 and Table 2 showed the physical properties of those aggregates tested according to the code GB/T 17431 [38] (similar to the code of ASTM C330) and JGJ 52 [39] (similar to the code of ASTM C33), respectively. The chopped CFs (Figure 3) with a length of 10 mm and a diameter of 7.3 μm were used in this study, which have an elastic modulus of 231 GPa, a tensile strength of 4558 MPa, an elongation at break of 2.05%, and a density of 1820 kg/m^3^. In order to obtain a good dispersion of CFs in the mixtures, hydroxypropyl methylcellulose (HPMC) and AGITAN P803 were used as dispersing agent and antifoaming agent, respectively. A QS-8020H Polycarboxylate Superplasticizer (SP) was used to enhance the workability. The mixing water was seawater taken from the sea in Guangxi Beibu Gulf.

### 2.2. Mix Proportions and Specimen Preparation

**Figure 4:**
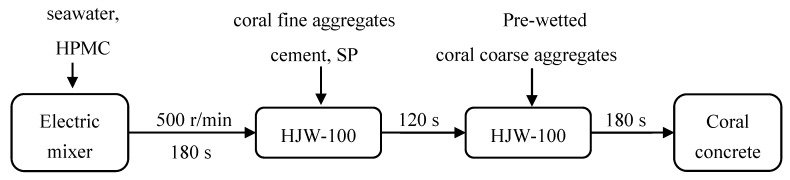
The process of the mixing method

The designed strength grades of CFRCC without CF addition were C20, C30, and C40, respectively. The basic mix proportions were designed according to JGJ 51 [40] and presented in Table 3. The CFs dosage were 0.0%, 0.3%, 0.6%, 1.0%, 1.5%, 2.0% by weight of the binder (cement). The usage of HPMC and P803 was 0.4% and 0.15% by weight of the binder, respectively. Some studies [13,41,42] pointed out that preparation of coral concrete with pre-wetted coral coarse aggregates is beneficial for improving the compressive strength, improving the workability, reducing the self-shrinkage and dry shrinkage of coral concrete. Thus, the coral coarse aggregates have been pre-wetted before mixing. The procedure of mixing CRFCC is illustrated in Figure 4. After the uniform mixture was obtained, the stirred mixture was cast in molds and vibrated for about 30 s on a vibration table. For each mixture, three 100 mm × 100 mm × 100 mm cubes and two 150 mm × 300 mm cylinders were cast. All cast specimens were cured at room temperature for 24 h, then demolded and cured in a marine environment curing cabinet for 28 d. The marine environment curing cabinet has a seawater spray device and several related sensors that can simulate the humidity of the real marine environment. Then, cube specimens conducted the cube compressive strength test following the code GB/T50081 [43] (similar to the code of ASTM C33, but the specimen used is cube specimen instead of cylindrical specimen) to obtain the cube compressive strength of each CFRCC mixtures (see Table 4), and each cylindrical specimen was cut into four discs of 150 mm × (63 ± 2) mm for the drop-weight impact test.

### 2.3. Impact Tests

**Figure 5:**
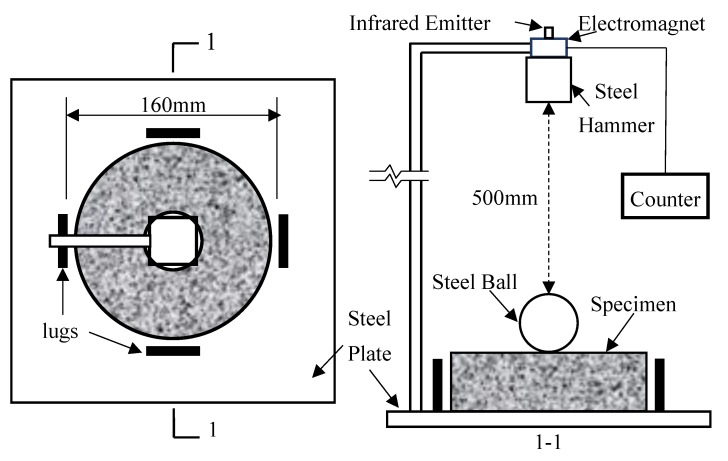
The details of the drop-weight impact test setup.

The impact test was conducted following the China CECS 13 [34] drop-weight impact test that was modified from the ACI 544 [44] suggested method. The details of the drop-weight impact test setup are illustrated in Figure 5. As shown in Figure 5, a steel hammer with a mass of 4.5 kg drops from a height of 500 mm on a steel ball with a diameter of 63 mm located on the central surface of the disc specimens. The number of blows causing the first visible crack was recorded as the initial crack resistance factor (*N_1_*), and the number of blows until the pieces of specimen touching three of the four steel lugs was recorded as the final failure resistance factor (*N_2_*). For each mixture, eight discs were tested, and the impact resistance was represented based on the average of eight specimens. The impact energy at initial crack and final failure were calculated by using the following equation:

(1) $$W_{i}=N_{i}\times \frac{1}{2}\times m\times v^{2}=N_{i}mgh,$$

where $W_{i}$ is the impact energy (J); $N_{i}$ is the number of blows; *m* is the weight of steel hammer with a mass of 4.5 kg; v is the velocity of the steel hammer (m/s); g is the acceleration of gravity (9.81 m/s^2^); h is the falling height of the steel hammer (500 mm); and *i* = 1, 2 is representing the initial crack and final failure, respectively.

## 3. Results and Discussion

### 3.1. Failure Patterns under Impact

**Figure 6:**
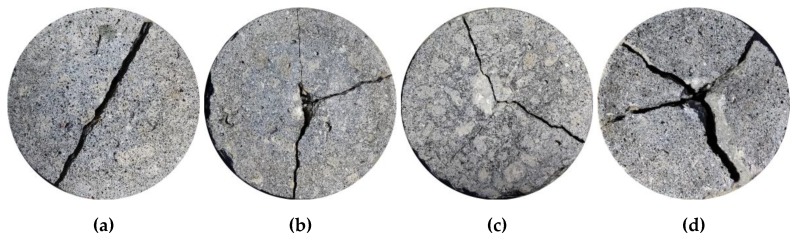
The failure patterns of part of specimens: (**a**) CC30C00; (**b**) CC30C06; (**c**) CC30C15; (**d**) CC20C20.

After drop-weight impact tests, the failure patterns of part of the specimens with and without CFs are shown in Figure 6. As expected, for all the specimens without CFs, when the first visible crack appears, the specimens suddenly broke down into two pieces and showed an obviously brittle failure behavior. For the specimens with CFs at a low level, its failure pattern is similar to the specimens without CFs, but some specimens broke down into three pieces (Figure 6b). For the specimens with CFs at a high level, after the first visible crack appears, the specimen can continue to bear the impact loads, and finally break into two or three or four pieces (Figure 6c,d). It is worth noting that no matter whether the dosage of CFs is high or low, the specimens will eventually be wholly separated into several parts, which is similar to the basalt fibers reinforced concrete [45] but different from the impact failure phenomenon—the specimen still remains intact—of steel fibers reinforced concrete, macro polypropylene fibers reinforced concrete, NiTi-SMA fibers reinforced concrete, and polypropylene fibers reinforced concrete [21,36,46]. The reason is that the diameter of CFs is only 7.3 μm, and the elongation at the break of CFs is no greater than 2.05%; when CFs are added into coral concrete, there are tens of millions of micro CFs that exist in the coral concrete matrix, and almost all the microcracks have micro CFs, which can restrain the microcracks propagation and hence enhance the impact performance of CFRCC at the microcrack stage, but many CFs have been broken or pulled out at macrocracks stage, so the CFs mainly act in the microcrack stage, and have less hindrance effect on large cracks. Moreover, with the strength grade and CF dosage increasing, a more profound impact pit and more debris were observed at the central surface of the specimen when the specimen fails.

**Figure 7:**
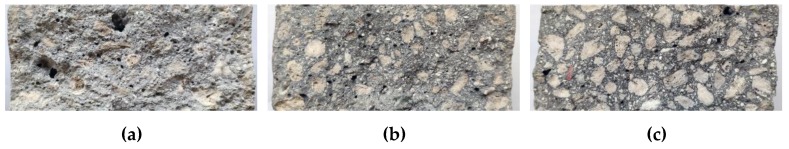
The fracture surface of part of specimens: (**a**) C20; (**b**) C30; (**c**) C40.

Figure 7 shows the fracture surface of part of the specimens after repeated drop-weight impact tests. It can be seen that, when the concrete strength grade is C20, there is a small amount of coral coarse aggregates broken (see Figure 7a), but almost all coral coarse aggregates broke (see Figure 7c) when the concrete strength grade is raised to C40. It can be concluded that the fracture rate of coral aggregates on the fracture surface increases with the increase of concrete strength grade. This phenomenon can be attributed to the relatively low strength of the coral coarse aggregates and the excellent bonding properties between the coral coarse aggregates and the cement matrix due to the rough surface morphology of the coral coarse aggregates [47].

### 3.2. Effect of CFs on the Impact Resistance

Table 5 summarizes the drop-weight impact test results for all the CFRCC mixtures (the detailed results of each specimen see Appendix A Table A1) where an increase in the number of post-first crack blow (*INPB*) is introduced, and the *INPB* is calculated as follows:

(2) $$INPB=N_{2}-N_{1},$$

where *N*_1_ and *N*_2_ are representing the number of blows at initial crack and final failure, respectively.

For the specimens of CC20C00, the first crack impact energy (*W_1_*) equals the failure impact energy (*W_2_*). For the specimens of CC30C00 and CC40C00, the failure impact energy (*W_2_*) is only 2 J and 6 J more than the first crack impact energy (*W_1_*). That is to say, when the first visible crack appears, the final failure of the specimen will occur at the same time, and the specimens without CFs show distinct brittle behavior.

**Figure 8:**
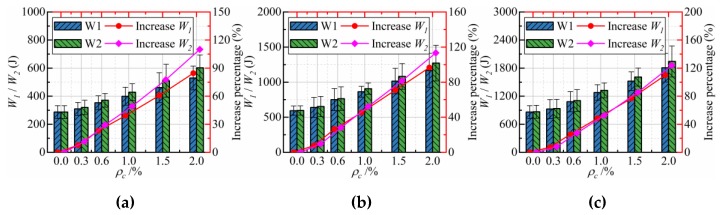
The impact energy at first crack and final failure of specimens with different CFs: (**a**) C20; (**b**) C30; (**c**) C40.

Figure 8 shows the effect of CFs dosage on the impact energy at first crack (*W_1_*) and final failure (*W_2_*) of CFRCC of three strength grades. It is easily found from the Figure 8 that adding CFs in coral concrete can improve the first impact energy and the final failure impact energy, and further improvement was recorded for the final failure impact energy, as compared to the first impact energy. With the increasing of additional CFs in coral concrete, the increase percentage of *W_1_* and *W_2_* is also increasing. In other words, the addition of CFs in coral concrete can improve both the initial crack and ultimate failure impact resistances of CFRCC, and its improvement increases with the increase of CF dosage.

**Figure 9:**
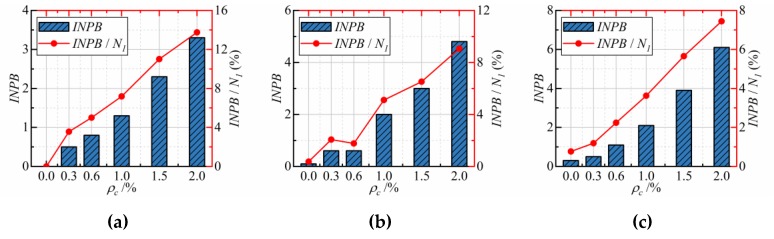
The *INPB* of specimens with different CFs: (**a**) C20; (**b**) C30; (**c**) C40.

Figure 9 exhibits the effect of CFs dosage on the *INPB* and *INPB*/*N_1_* of three strength grades’ CFRCC. In Figure 9, there is a clear trend of *INPB* and *INPB*/*N_1_* increasing with the increasing of CFs dosage. With the increasing of concrete strength grade, the *INPB* is also increasing while the *INPB*/*N_1_* decreases. It must be noted that, even with a CF dosage of 2.0%, *INPB* is also small, only 3.3, 4.8, and 6.1 for C20, C30, and C40, respectively, and the *INPB*/*N_1_* for all the mixture is no more than 14%. Mastali et al. [27] conducted the drop-weight impact test on CF reinforced self-compacting concrete and obtained similar results. That is to say, the improvement effect of CFs on the impact resistance of specimens after cracking is not apparent, which is obviously different from the test results of steel fibers reinforced concrete and macro PP fibers reinforced concrete obtained by Zhang, Rahmani, Ding, and Murali et al. [36,48,49,50]. The explanation for this is that the steel fibers are macro fibers (the diameter is generally higher than 0.4 mm) and have a relatively large elongation at break (more than 3.5%), so the steel fibers can play an excellent bridging role in macrocracks after the first visible crack appeared of specimens. However, the CFs have a diameter of only 7.3 μm and an elongation at break of only 2.05%, so the CFs mainly play a positive role in microcracks and a less positive role in macrocracks under the drop-weight impact test. The previous research data [26,50,51] also clearly indicated that, in the drop-weight impact test, the larger the diameter of fiber and elongation at break is, the larger the *INPB* will be, when other conditions are the same.

### 3.3. Effect of Concrete Strength Grade on the Impact Resistance

**Figure 10:**
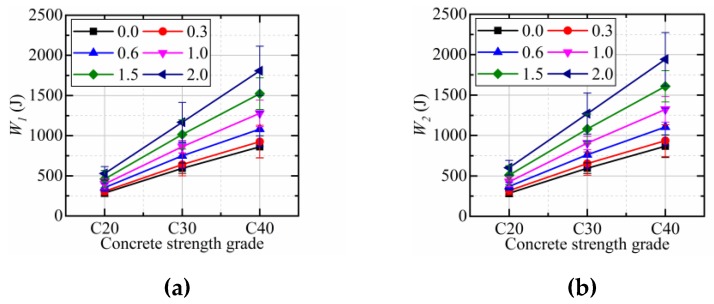
Comparison of test value and predicted value of splitting tensile strength: (**a**) *W_1_*; (**b**) *W_2_*.

As shown in Figure 10, the impact energy at first crack (*W_1_*) and final failure (*W_2_*) and strength grade is approximately in a linear relationship, which indicates that, for CFRCC, the higher the concrete strength grade is, the higher the impact resistance will be. For polypropylene fibers reinforced coral concrete, Wang et al. [21] also reached a similar conclusion.

### 3.4. Correlation between Cube Compressive Strength, CFs Dosage, and Impact Energy

After regression analysis, it is found that the effect of CFs dosage and cube compressive strength on the impact resistance of CFRCC can be illustrated by Equation (3):

(3) $$W_{1}\left(W_{2}\right)=\left(a+b\;{f_{cu}}^{1.5}\right)\left(c+d\;{\rho_{c}}^{1.2}\right),$$

where *W_1_ and W*_2_ are the impact energy at the first visible crack and final failure, respectively (J); *f_cu_* is the cube compressive strength (MPa); *ρ_c_* is the CFs dosage (%); *a*, *b*, *c,* and *d* are fitting parameters.

**Figure 11:**
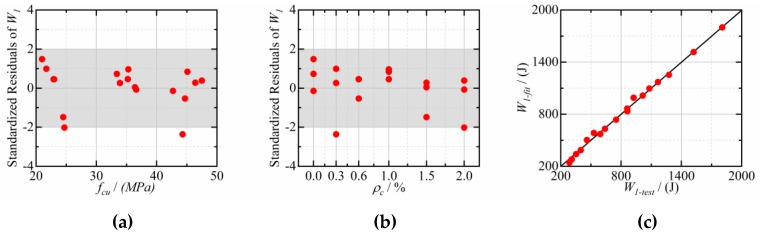
The fitting results of *W_1_*: (**a**) Standardized Residuals vs. *f_cu_*; (**b**) Standardized Residuals vs. *ρ_c_*; (**c**) comparison of test and fitting.

**Figure 12:**
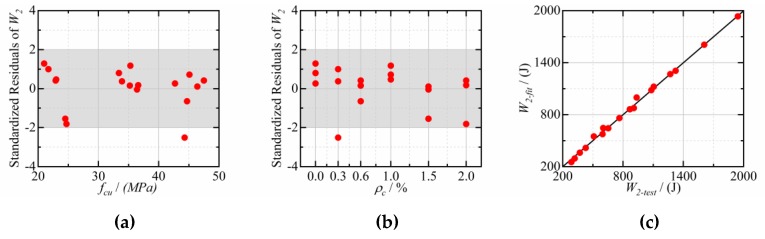
The fitting results of *W_2_*: (**a**) Standardized Residuals vs. *f_cu_*; (**b**) Standardized Residuals vs. *ρ_c_*; (**c**) comparison of test and fitting.

The fitting results of Equation (3) to test data are presented in Table 6, Figure 11, and Figure 12. It can be seen that the standardized residuals of most of the points are in the range of −2 to 2, and the Adjusted *R*^2^ are 0.995 and 0.996 for *W_1_* and *W_2_*, respectively, which indicates that Equation (3) fits the experimental data well. Moreover, Figure 11c and Figure 12c also indicate that the fitting values are very close to the experimental values.

## 4. Distribution of Impact Resistance Factors

Over the past few decades, several statistical models have been employed for analysis of the variations in impact test results of concrete [36,46,49,50,52,53,54,55,56]. Among them, the normal distribution model is widely used. However, many researchers [54,56] pointed out that the impact test results exhibited poor fitness with normal distribution at a 95% confidence level. By contrast, the two-parameter Weibull distribution has been proved by some researchers [36,46,50] that it is appropriate to evaluate the impact performance of concrete under impact. Therefore, for analyzing the variations in the impact resistance of CFRCC under drop-weight impact test, the two-parameter Weibull distribution is employed in this study.

According to [46], the expression of the cumulative distribution function *F(x)* of two-parameter Weibull probability law is as follows:

(4) $$F\left(x\right)=1-\exp\left[-{\left(\frac{x-x_{0}}{\lambda }\right)}^{k}\right],$$

where x is the impact life of the concrete; k is the shape parameter; λ is the scale parameter; x0 is the minimum impact life of concrete and assumed to be 0 in this study.

The function *F(x)* denotes the failure probability of concrete under impact loading. Thus, the probability estimator *L(x)* may be defined as:

(5) $$L\left(x\right)=1-F\left(x\right)=\exp\left[-{\left(\frac{x-x_{0}}{\lambda }\right)}^{k}\right],$$

Take *x_0_* = 0 and the natural logarithm twice on both sides of Equation (5) to get:

(6) $$\ln\ln\frac{1}{L\left(x\right)}=k\ln x-k\ln\lambda .$$

Thus, Equation (6) can be used to verify whether the impact resistance factors (*N_1_*, and *N_2_*) of CFRCC follow the two-parameter Weibull distribution. Since Equation (6) represents a linear relationship between ln ln (1/*L(x)*) and ln *x*, if an appropriately linear relationship between ln ln (1/*L(x)*) and ln *x* is observed from the test results, the conclusion that using two-parameter Weibull distribution to characterize the statistical distribution of impact test results of CFRCC is feasible can be conducted. In order to verify whether there is an appropriately linear relationship between ln (1/*L(x)*) and ln *x*, first, the impact results (*N_1_*, and *N_2_*) are arranged in an descending order, and then the probability estimator is assumed and the linear regression analysis is performed.

Many probability estimators have been used in previous studies and Murali et al. [50] summarized twenty probability estimators used in previous papers. It can be seen from the summaries of Murali [50] that there are two expression forms of the probability estimator:

(7) $$L\left(x\right)=\frac{j+\alpha }{n+\beta },$$

(8) $$L\left(x\right)=1-\frac{j+\alpha }{n+\beta },$$

where *j* is the sequence number of the impact failure specimen; *n* is the total number of the impact specimens for each mixture; *α* and *β* are constants.

**Figure 13:**
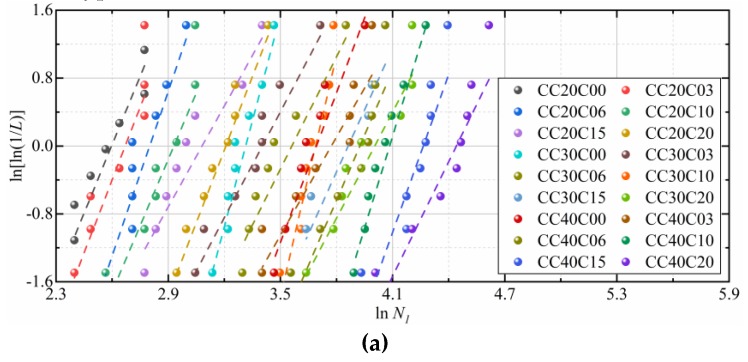

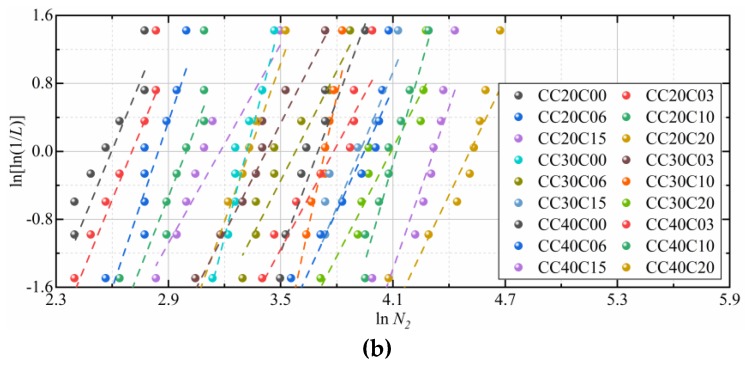
Linear regression of *N_1_* and *N_2_* in Weibull distribution: (**a**) *N_1_*; (**b**) *N_2_*.

After trial calculating the test results with Equations (7) and (8), Equation (7) is chosen as the recommended probability estimator in this study, and the values of *α* and *β* are −0.6, and 0.9, respectively. Figure 13 shows the distribution of the impact resistance factor (*N_1_*, and *N_2_*) of each CFRCC mixture and the corresponding fitted curves, and Table 7 gives the detailed linear regression results. Rahmani et al. [49] pointed out that a *R*^2^ of 0.7 or higher is sufficient for establishing a reasonable reliability model. Since the appropriately linear relationship plot in Figure 13 and all the impact test results have Adjusted *R*^2^ equal to or higher than 0.837, the two-parameter Weibull distribution is considered suitable for establishing the statistical distribution of impact test data of coral concrete incorporating CFs. These developed reliability curves are highly suitable as a useful tool to quickly investigate the impact resistance of CFRCC, thereby eliminating the necessity of time-consuming impact testing process. Some previous studies [36,46,49,50] have drawn similar conclusions for other types of fibers reinforced concrete.

According to Equations (5) and (6), the number of blows (*N_1_, N_2_*) of CFRCC at the corresponding failure probability *P* can be derived as follows:

(9) $$N=x=\frac{1}{k}\ln\ln\frac{1}{1-P}+\ln\lambda ,$$

where *P* is the failure probability.

**Figure 14:**
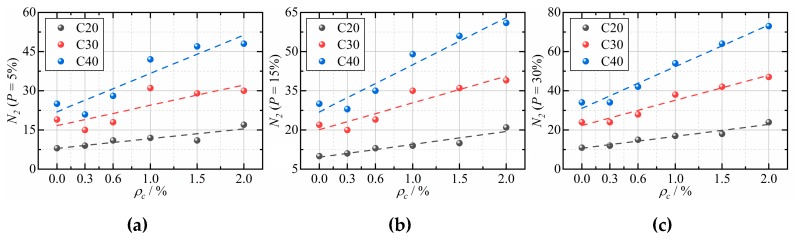
*N_2_* vs. *ρ_c_* based on reliability analysis with different failure probability: (**a**) *P* = 5%; (**b**) *P* = 15%; (**c**) *P* = 30%.

Figure 14 shows the *N_2_* of CFRCC acquired by reliability analysis at different failure probability. It is easy to note that the impact resistance performance of CFRCC increases approximately linearly with the CF dosage increasing at the same failure probability.

As an example of verifying whether the two-parameter Weibull distribution recommended in this study is also suitable to evaluate the impact performance of other fibers reinforced concrete, the test results of Ding et al. [36] for macro polypropylene fibers and steel fibers reinforced concrete are also analyzed by using Equations (6) and (7), and the regression analysis results are given in Table 8. From Table 8, it can be seen that the Adjusted R^2^ of each mixture is no less than 0.833, which indicates that the two-parameter Weibull distribution recommended in this study is also suitable to evaluate the impact performance of other types of fibers reinforced concrete.

## 5. Conclusions

In this study, the impact resistance of CFRCC under impact loading was investigated by conducting the drop-weight impact test. Based on the experimental results and regression analysis, the main conclusions can be drawn as follows:

(1) The addition of CFs into coral concrete changed the failure pattern of coral concrete specimens under impact loading from obvious brittleness to relatively good ductility.
(2) CF addition can improve the impact resistance at initial crack and final failure of coral concrete. Still, the improvement of the impact resistance after initial cracking due to the addition of CFs is not as significant as steel fibers.
(3) The impact resistance of CFRCC increases with the increase of CF dosage and concrete strength grade.
(4) The impact energy (*W_1_*, and *W_2_*) of CFRCC can be evaluated by the cube compressive strength and CFs dosage using Equation (3).
(5) The two-parameter Weibull distribution theory is proved capable of adequately representing the impact test results, and these developed reliability curves through the two-parameter Weibull distribution theory can be considered a useful tool to investigate the impact resistance of CFRCC quickly.

## Figures and Tables

**Table 1:** Physical properties of coral coarse aggregates.

Bulk Density (kg/m^3^)	Apparent Density (kg/m^3^)	Void Content (%)	Water Absorption/%		Water Content (%)	Tube Compressive Strength (MPa)	Dust Content (%)	Particle Shape Factor
			1 h	24 h				
915	1841	50	8.5	11.0	2.6	3.1	2.9	2.4

**Table 2:** Physical properties of coral fine aggregates.

Bulk Density (kg/m^3^)	Apparent Density (kg/m^3^)	Graduation	Fineness Modulus	Water Content (%)	Water Absorption (%)	Dust Content
1296	2707	II	3.0	2.9	3.7	0.5

**Table 3:** Coral concrete mix proportions

Strength Grade	Cement (kg)	Net W/C^1^	Net Water (kg)	Additional Water (kg)	Coral Coarse Aggregates (kg)	Coral Sand (kg)
CC20C00	380	0.53	200	5.8	774	674
CC30C00	480	0.38	180	5.8	774	674
CC40C00	650	0.28	180	5.8	774	674

^1^ Net *W/C*= Net water/Cement.

**Table 4:** Cube compressive strength.

No.	Average (MPa)	Standard Deviation	Coefficient of Variation
CC20C00	21.0	3.08	0.15
CC20C03	21.7	1.83	0.08
CC20C06	22.9	1.34	0.06
CC20C10	23.0	2.02	0.09
CC20C15	24.5	2.12	0.09
CC20C20	24.7	0.42	0.02
CC30C00	33.4	0.91	0.03
CC30C03	33.9	2.52	0.07
CC30C06	35.2	3.56	0.10
CC30C10	35.3	1.56	0.04
CC30C15	36.4	1.39	0.04
CC30C20	36.6	4.69	0.13
CC40C00	42.7	4.91	0.11
CC40C03	44.3	2.16	0.05
CC40C06	44.7	1.04	0.02
CC40C10	45.1	2.03	0.05
CC40C15	46.4	2.77	0.06
CC40C20	47.5	2.59	0.05

**Table 5:** The drop-weight impact tests results.

No.	Average Number of Blows			Standard Deviation			Coefficient of Variation			$W_{1}(J)$	$W_{2}(J)$
	$N_{1}$	$N_{2}$	INPB	$\sigma_{N_{1}}$	$\sigma_{N_{2}}$	$\sigma_{INPB}$	$CV_{N_{1}}$	$CV_{N_{2}}$	$CV_{INPB}$		
CC20C00	13.0	13.0	0.0	2.14	2.14	0.00	0.16	0.16	0.00	287	287
CC20C03	14.0	14.5	0.5	2.07	2.33	0.79	0.15	0.16	1.59	309	320
CC20C06	16.0	16.8	0.8	2.20	2.19	0.45	0.14	0.13	0.60	353	371
CC20C10	18.1	19.4	1.3	2.90	2.77	0.34	0.16	0.14	0.27	400	428
CC20C15	20.9	23.1	2.3	4.85	5.41	1.26	0.23	0.23	0.56	461	510
CC20C20	24.0	27.3	3.3	3.85	3.99	0.84	0.16	0.15	0.26	530	603
CC30C00	26.9	27.0	0.1	2.95	2.88	0.28	0.11	0.11	2.26	594	596
CC30C03	29.0	29.6	0.6	6.37	6.39	0.77	0.22	0.22	1.22	640	653
CC30C06	34.0	34.6	0.6	7.17	7.46	0.81	0.21	0.22	1.29	750	764
CC30C10	39.1	41.1	2.0	3.68	3.56	0.95	0.09	0.09	0.48	863	907
CC30C15	46.0	49.0	3.0	8.23	8.18	0.70	0.18	0.17	0.23	1015	1082
CC30C20	52.9	57.6	4.8	11.26	11.71	2.58	0.21	0.20	0.54	1168	1271
CC40C00	39.0	39.3	0.3	6.21	6.11	0.36	0.16	0.16	1.46	861	867
CC40C03	41.9	42.4	0.5	9.20	8.93	0.48	0.22	0.21	0.96	925	936
CC40C06	49.0	50.1	1.1	9.99	10.47	1.12	0.20	0.21	1.00	1082	1106
CC40C10	57.9	60.0	2.1	7.43	7.17	1.09	0.13	0.12	0.51	1278	1324
CC40C15	69.0	72.9	3.9	8.94	9.08	1.26	0.13	0.12	0.33	1523	1609
CC40C20	81.9	88.0	6.1	13.88	14.59	1.83	0.17	0.17	0.30	1808	1942

**Table 6:** Fitting results.

Dependent Variable	a	b	c	d	Adjusted R^2^
$W_{1}$	−32.035	1.306	2.601	0.848	0.995
$W_{2}$	−26.791	1.326	2.510	0.975	0.996

**Table 7:** Linear regression results of impact resistance in Weibull distribution.

Specimen No.	*N_1_*			*N_2_*		
	*k*	*λ*	Adjusted R^2^	k	λ	Adjusted R^2^
CC20C00	5.382	13.420	0.853	5.382	13.420	0.853
CC20C03	5.723	14.437	0.874	5.375	14.964	0.926
CC20C06	6.468	16.454	0.865	6.514	17.227	0.864
CC20C10	5.190	18.730	0.884	5.593	20.001	0.841
CC20C15	3.971	21.779	0.881	3.910	23.983	0.894
CC20C20	5.736	24.720	0.962	6.196	28.027	0.982
CC30C00	8.318	27.497	0.924	8.507	27.615	0.919
CC30C03	4.173	30.040	0.946	4.302	30.667	0.950
CC30C06	4.410	35.203	0.888	4.298	35.869	0.885
CC30C10	9.444	39.945	0.940	10.241	41.929	0.956
CC30C15	4.874	47.590	0.849	5.302	50.598	0.858
CC30C20	4.179	54.810	0.912	4.259	59.766	0.869
CC40C00	5.878	40.164	0.895	5.954	40.420	0.871
CC40C03	3.862	43.493	0.908	4.071	43.957	0.927
CC40C06	5.112	50.648	0.902	4.783	51.757	0.897
CC40C10	7.251	59.354	0.942	7.689	61.472	0.938
CC40C15	6.201	71.067	0.848	6.363	75.026	0.837
CC40C20	4.503	84.860	0.902	4.579	91.076	0.885

**Table 8:** Linear regression results of impact resistance of Ding et al.’s [36] results in Weibull distribution.

Specimen No.	*N_1_*			*N_2_*		
	*k*	*λ*	Adjusted R^2^	*λ*	*k*	Adjusted R^2^
NC	1.243	11.674	0.927	1.243	11.674	0.927
PP4	2.187	15.109	0.712	2.726	30.833	0.865
PP6	6.892	21.914	0.958	5.357	38.456	0.952
SF20	1.134	22.016	0.943	1.352	41.712	0.958
SF35	0.860	19.937	0.988	1.389	45.208	0.899

## Appendix A

**Table A1:** The detail drop-weight impact test results of each specimen.

No.		1	2	3	4	5	6	7	8	Mean	Standard Deviation	Coefficient of Variation
CC20C00	$N_{1}$	11	12	16	11	16	14	11	13	13.0	2.14	0.16
	$N_{2}$	11	12	16	11	16	14	11	13	13.0	2.14	0.16
	INPB	0	0	0	0	0	0	0	0	0.0	0.00	0.00
CC20C03	$N_{1}$	16	11	16	16	12	12	14	15	14.0	2.07	0.15
	$N_{2}$	16	11	17	17	12	13	14	16	14.5	2.33	0.16
	INPB	0	0	1	1	0	1	0	1	0.5	0.79	1.59
CC20C06	$N_{1}$	13	17	15	20	15	15	15	18	16.0	2.20	0.14
	$N_{2}$	13	18	16	20	16	16	16	19	16.8	2.19	0.13
	INPB	0	1	1	0	1	1	1	1	0.8	0.45	0.60
CC20C10	$N_{1}$	13	17	17	19	21	21	21	16	18.1	2.90	0.16
	$N_{2}$	14	19	18	20	22	22	22	18	19.4	2.77	0.14
	INPB	1	2	1	1	1	1	1	2	1.3	0.34	0.27
CC20C15	$N_{1}$	18	18	18	19	17	27	30	20	20.9	4.85	0.23
	$N_{2}$	20	20	20	21	18	30	33	23	23.1	5.41	0.23
	INPB	2	2	2	2	1	3	3	3	2.3	1.26	0.56
CC20C20	$N_{1}$	22	26	19	25	20	23	26	31	24.0	3.85	0.16
	$N_{2}$	25	29	21	28	24	27	30	34	27.3	3.99	0.15
	INPB	3	3	2	3	4	4	4	3	3.3	0.84	0.26
CC30C00	$N_{1}$	32	28	30	25	25	23	26	26	26.9	2.95	0.11
	$N_{2}$	32	28	30	25	26	23	26	26	27.0	2.88	0.11
	INPB	0	0	0	0	1	0	0	0	0.1	0.28	2.26
CC30C03	$N_{1}$	33	29	26	22	30	30	41	21	29.0	6.37	0.22
	$N_{2}$	34	29	27	24	30	30	42	21	29.6	6.39	0.22
	INPB	1	0	1	2	0	0	1	0	0.6	0.77	1.22
CC30C06	$N_{1}$	28	42	32	36	29	27	31	47	34.0	7.17	0.21
	$N_{2}$	29	43	32	37	29	27	32	48	34.6	7.46	0.22
	INPB	1	1	0	1	0	0	1	1	0.6	0.81	1.29
CC30C10	$N_{1}$	33	42	37	44	43	38	37	39	39.1	3.68	0.09
	$N_{2}$	35	43	38	46	44	42	39	42	41.1	3.56	0.09
	INPB	2	1	1	2	1	4	2	3	2.0	0.95	0.48
CC30C15	$N_{1}$	39	38	38	58	54	40	53	48	46.0	8.23	0.18
	$N_{2}$	42	41	42	62	57	43	55	50	49.0	8.18	0.17
	INPB	3	3	4	4	3	3	2	2	3.0	0.70	0.23
CC30C20	$N_{1}$	51	44	38	67	46	63	67	47	52.9	11.26	0.21
	$N_{2}$	53	51	41	72	50	71	70	53	57.6	11.71	0.20
	INPB	2	7	3	5	4	8	3	6	4.8	2.58	0.54
CC40C00	$N_{1}$	36	38	32	42	52	41	34	37	39.0	6.21	0.16
	$N_{2}$	36	38	33	42	52	42	34	37	39.3	6.11	0.16
	INPB	0	0	1	0	0	1	0	0	0.3	0.36	1.46
CC40C03	$N_{1}$	48	30	54	40	49	48	30	36	41.9	9.20	0.22
	$N_{2}$	48	32	54	41	49	49	30	36	42.4	8.93	0.21
	INPB	0	2	0	1	0	1	0	0	0.5	0.48	0.96
CC40C06	$N_{1}$	28	55	51	49	41	55	55	58	49.0	9.99	0.20
	$N_{2}$	28	57	52	50	42	56	57	59	50.1	10.47	0.21
	INPB	0	2	1	1	1	1	2	1	1.1	1.12	1.00
CC40C10	$N_{1}$	72	58	55	53	64	60	49	52	57.9	7.43	0.13
	$N_{2}$	73	59	59	56	66	63	52	52	60.0	7.17	0.12
	INPB	1	1	4	3	2	3	3	0	2.1	1.09	0.51
CC40C15	$N_{1}$	74	51	72	81	74	70	65	65	69.0	8.94	0.13
	$N_{2}$	78	54	75	84	79	74	68	71	72.9	9.08	0.12
	INPB	4	3	3	3	5	4	3	6	3.9	1.26	0.33
CC40C20	$N_{1}$	92	83	67	87	85	90	96	55	81.9	13.88	0.17
	$N_{2}$	99	91	73	93	91	96	102	59	88.0	14.59	0.17
	INPB	7	8	6	6	6	6	6	4	6.1	1.83	0.30
